# *Arabidopsis* ABA Receptor RCAR1/PYL9 Interacts with an R2R3-Type MYB Transcription Factor, AtMYB44

**DOI:** 10.3390/ijms15058473

**Published:** 2014-05-13

**Authors:** Dekuan Li, Ying Li, Liang Zhang, Xiaoyu Wang, Zhe Zhao, Zhiwen Tao, Jianmei Wang, Jin Wang, Min Lin, Xufeng Li, Yi Yang

**Affiliations:** 1 Key Laboratory of Bio-Resources and Eco-Environment of Ministry of Education, College of Life Sciences, Sichuan University, Chengdu 610065, China; E-Mails: lidekuan198621@126.com (D.L.); lyscholes@163.com (Y.L.); liang.zhang113@gmail.com (L.Z.); ccandy1021@163.com (X.W.); zhaozhe89@126.com (Z.Z.); scuzhiwen8508@126.com (Z.T.); wangjianmei@scu.edu.cn (Jia.W.); lixufeng0507@gmail.com (X.L.); 2 State Key Laboratory of Hydraulics and Mountain River Engineering, Sichuan University, Chengdu 610065, China; 3 Biotechnology Research Institute, Chinese Academy of Agricultural Sciences/Key Laboratory of Crop Biotechnology, Ministry of Agriculture, Beijing 100081, China; E-Mails: wjdsz@vip.sina.com (JinW.); linmin57@vip.163.com (M.L.)

**Keywords:** ABA, signaling, RCAR1/PYL9, receptor, AtMYB44, ABI1

## Abstract

Abscisic acid (ABA) signaling plays important roles in plant growth, development and adaptation to various stresses. RCAR1/PYL9 has been known as a cytoplasm and nuclear ABA receptor in *Arabidopsis*. To obtain further insight into the regulatory mechanism of RCAR1/PYL9, a yeast two-hybrid approach was performed to screen for RCAR1/PYL9-interacting proteins and an R2R3-type MYB transcription factor, AtMYB44, was identified. The interaction between RCAR1/PYL9 and AtMYB44 was further confirmed by glutathione S-transferase (GST) pull-down and bimolecular fluorescence complementation (BiFC) assays. Gene expression analysis showed that AtMYB44 negatively regulated the expression of ABA-responsive gene *RAB18*, in contrast to the opposite role reported for RCAR1/PYL9. Competitive GST pull-down assay and analysis of phosphatase activity demonstrated that AtMYB44 and ABI1 competed for binding to RCAR1/PYL9 and thereby reduced the inhibitory effect of RCAR1/PYL9 on ABI1 phosphatase activity in the presence of ABA *in vitro*. Furthermore, transient activation assay in protoplasts revealed AtMYB44 probably also decreased RCAR1/PYL9-mediated inhibition of ABI1 activity *in vivo.* Taken together, our work provides a reasonable molecular mechanism of AtMYB44 in ABA signaling.

## 1. Introduction

As sessile organisms, plants cannot escape away from, but have to face environmental stresses such as heat, cold, drought, and high-salinity stresses [1,2]. The phytohormone abscisic acid (ABA) is the crucial regulator in plant response to these stresses [3]. ABA enhances the capacity of plants in response to stress conditions through inducing stomatal closure, changing gene expression and increasing the accumulation of osmo-compatible solutes [3,4].

In the last several years, the core ABA signaling components have been well ascertained. The RCARs/PYR1/PYLs act as ABA receptors [5–8], and the type 2C protein phosphatases (PP2Cs) act as negative regulators [9], while the SNF1-related protein kinase 2 family (SnRK2s) act as positive regulators [10–12]. In the absence of ABA, the PP2Cs interact with and dephosphorylate SnRK2s to repress their kinase activities [3,13]. In the presence of ABA, RCARs/PYR1/PYLs interact with PP2Cs and inhibit their phosphatase activities, allowing SnRK2s to be activated [3,13]. Then, activated SnRK2s directly phosphorylate transcription factors ABFs/AREBs or components of the machinery regulating stomatal aperture, leading to ABA responses [3,13].

Besides these core ABA signaling elements described above, MYB transcription factors (MYBs) are also key components in ABA signaling [14]. For example, AtMYB44, an R2R3-MYB transcription factor, has been well characterized for their regulatory roles in response to ABA [15–18]. Interestingly, controversial issues occurred about these studies. Huang *et al*. [15] and Jaradat *et al*. [16] demonstrated that AtMYB44 negatively regulated ABA signaling, while Jung *et al*. [17], and Persak and Pitzschke [18] revealed that over-expression of *AtMYB44* led to ABA hypersensitivity in *Arabidopsis*. In addition, AtMYB44 was shown to be phosphorylated by MPK3 [18,19], but how AtMYB44 participates in ABA signaling remains unknown.

In this study, we demonstrate that AtMYB44 interacts with the ABA receptor RCAR1/PYL9, and reduces its inhibitory effect on ABI1 activity. This finding indicates that AtMYB44 may act as a negative regulator in ABA signaling. To our knowledge, this is the first detailed report on the molecular mechanism of how AtMYB44 is involved in ABA signaling regulation.

## 2. Results

### 2.1. Identification of RCAR1/PYL9-Interacting Proteins by Yeast Two-Hybrid Screening

The yeast two-hybrid system provides an efficient means to characterize core components in ABA signaling. The ABA receptor RCAR1/PYL9 has been identified by this system using ABI2 as bait [5]. Here, we used the yeast two-hybrid system to identify proteins that interact with RCAR1/PYL9. As a result, several different types of proteins were identified (Table S1). Three of them are closely related members of MYBs, namely AtMYB44, AtMYB70 and AtMYB77 (Table 1). AtMYB44 was shown to be involved in ABA signaling [15–17]. Thus, in the present study, we mainly focused our study on the interaction between RCAR1/PYL9 and AtMYB44 and the significance of their interaction in ABA signaling based on the biochemical and molecular analysis.

**Figure 1.**
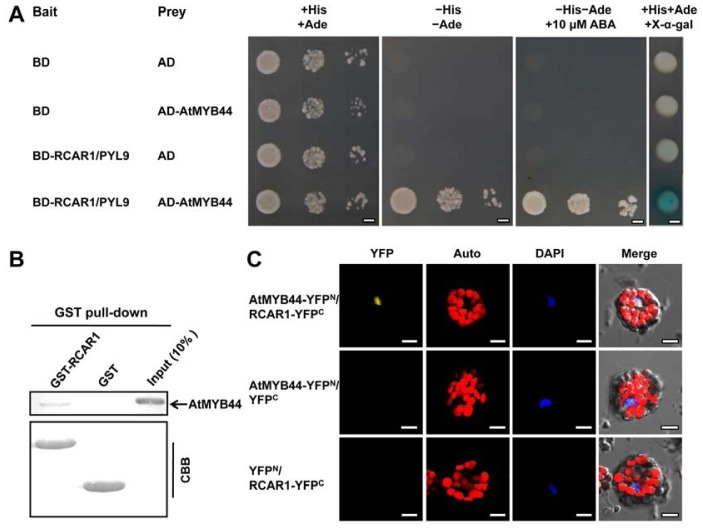
Physical interaction between AtMYB44 and RCAR1/PYL9 *in vitro* and *in vivo*. (**A**) Yeast two-hybrid assay. The interaction between AtMYB44 and RCAR1/PYL9 was determined by growth assay and X-α-gal staining on the indicated medium. When indicated, the medium was supplemented with 10 μM ABA. Dilutions (0.1, 0.01, and 0.001) (**left three panels**) or dilution (0.1) (**right panel**) of saturated yeast cultures containing the indicated plasmids were spotted on the indicated plates, and photographs were taken after 3 days at 30 °C. BD (for DNA binding domain) indicates pGBKT7 vector; AD (for transcription activation domain) indicates pGADT7 vector. Scale bars = 2.5 mm; (**B**) GST pull-down assay. 5 μg of recombinant His-AtMYB44 protein was incubated with GST or GST-RCAR1/PYL9 in GST affinity resin. The eluates were resolved by SDS-PAGE and blotted using anti-His antibody. The position of His-AtMYB44 is indicated by an arrow. CBB (Coomassie Brilliant Blue) staining indicates amounts of GST-tagged proteins. Three independent experiments were performed and similar results were obtained; and (**C**) BiFC assay in *Arabidopsis* protoplasts. The *N*-terminal half of YFP was fused to AtMYB44 (AtMYB44-YFP^N^), and the *C*-terminal half of YFP was fused to RCAR1/PYL9 (RCAR1-YFP^C^). The combinations of plasmids are shown on the left. Co-expression of AtMYB44-YFP^N^ and YFP^C^ or YFP^N^ and RCAR1-YFP^C^ was used as a negative control, respectively. The nuclei were stained by DAPI. Scale bars = 10 μm.

To test whether the full-length AtMYB44 and RCAR1/PYL9 interact, additional yeast two-hybrid assays were performed. As is shown in Figure 1A, AH109 yeast cells co-transformed with the full-length AtMYB44 prey construct and RCAR1/PYL9 bait vector grew well on SD/-L-T-H-A (–His–Ade) medium and turned blue on SD/-L-T (+His+Ade) + X-α-Gal medium compared with the control transformants, indicating that the full-length AtMYB44 indeed interacts with RCAR1/PYL9 in yeast. Considering that RCAR1/PYL9 is an ABA receptor and its conformation can be changed upon binding to ABA [3,13], we analyzed whether ABA affected the interaction between AtMYB44 and RCAR1/PYL9. Yeast two-hybrid assay showed that ABA had no significant effect on the interaction between these two proteins (Figure 1A).

### 2.2. Demonstration of the Physical Interaction between RCAR1/PYL9 and AtMYB44 in Vitro and in Vivo

To further confirm the interaction between RCAR1/PYL9 and AtMYB44, we performed *in vitro* GST pull-down assays. In these analyses, recombinant GST or GST-tagged RCAR1/PYL9 proteins were used to pull down His-tagged AtMYB44 proteins. As a result, AtMYB44 was retained by GST-RCAR1/PYL9 but not GST, suggesting that RCAR1/PYL9 interacts with AtMYB44 *in vitro* (Figure 1B). Subsequently, BiFC assay was employed to test the interaction between RCAR1/PYL9 and AtMYB44 *in planta*. AtMYB44 and RCAR1/PYL9 were fused to the *N*- and *C*-terminal region of YFP to form AtMYB44-YFP^N^ and RCAR1-YFP^C^, respectively, and the two constructs were co-transfected into *Arabidopsis* protoplasts. The empty vectors (YFP^C^ and YFP^N^) in combination with AtMYB44-YFP^N^ or RCAR1-YFP^C^ were used as negative controls. As a result, YFP fluorescence was observed in the nucleus of the protoplast cells co-transformed by AtMYB44-YFP^N^ and RCAR1-YFP^C^, while the negative controls failed to yield any fluorescent signal (Figure 1C). This result suggests that AtMYB44 interacts with RCAR1/PYL9 in the nucleus of *Arabidopsis* cells.

### 2.3. Analyses of the Interactions between RCARs/PYR1/PYLs and Other Members of the 22nd Subgroup of R2R3-MYBs

**Figure 2.**
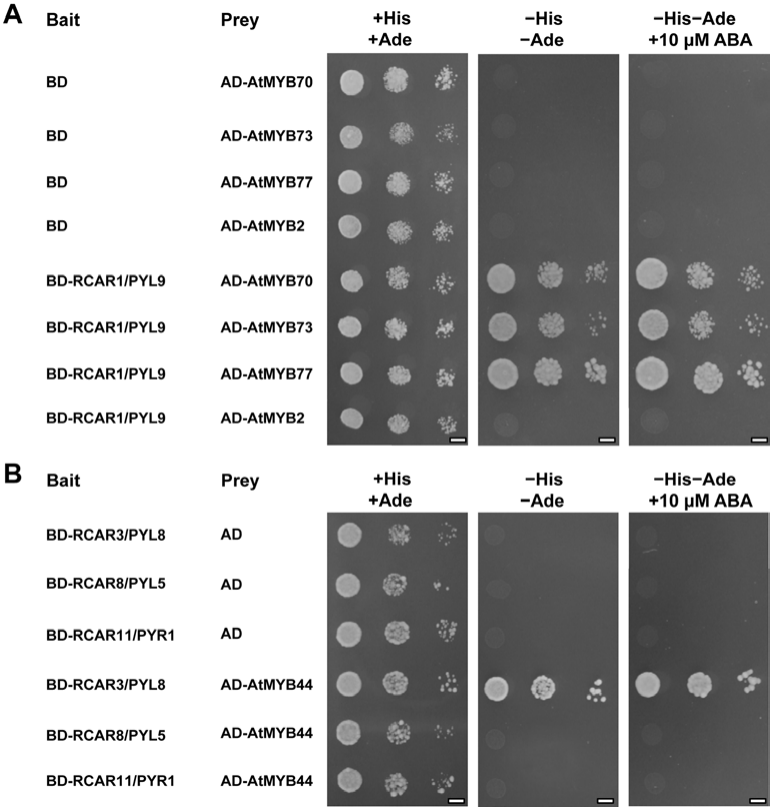
Interactions between RCARs/PYR1/PYLs and the other members of 22nd subgroup of R2R3-MYBs. (**A**) Yeast two-hybrid assay was performed using RCAR1/PYL9 as bait and the full-length AtMYB70, AtMYB73, and AtMYB77 and AtMYB2 as preys. The empty BD and prey vectors were used as negative controls. Picture of the plates were taken after 3 days at 30 °C; and (**B**) Yeast two-hybrid assay was performed using the full-length RCAR3/PYL8, RCAR8/PYL5 and RCAR11/PYR1 as baits and AtMYB44 as prey. The empty AD and bait vectors were used as negative controls. Pictures of the plates were taken after 3 days at 30 °C. Scale bars = 2.5 mm.

Considering that AtMYB44 belongs to the 22nd subgroup of R2R3-MYBs [20], we tested whether RCAR1/PYL9 also interacted with other members of the 22nd subgroup. Thus, AtMYB70, AtMYB73, AtMYB77 and AtMYB2 (as a control) were tested for their potential interactions with RCAR1/PYL9 in yeast two-hybrid assay. Figure 2A showed that RCAR1/PYL9 also interacted with AtMYB70, AtMYB73 and AtMYB77, but not with AtMYB2, a member of another subgroup of R2R3-MYBs, indicating that RCAR1/PYL9 may specially interact with the 22nd subgroup of MYBs. Similarly, ABA did not significantly affect the interactions between these proteins (Figure 2A).

RCARs/PYR1/PYLs have been grouped into three different classes in *Arabidopsis* and RCAR1/PYL9 belongs to class I [5]. To test whether AtMYB44 also interacts with the other two classes of RCARs/PYR1/PYLs, RCAR3/PYL8, RCAR8/PYL5 and RCAR11/PYR1 were chosen for representative of different classes. Yeast two-hybrid assays demonstrated that AtMYB44 only interacted with RCAR3/PYL8, but not with RCAR8/PYL5 and RCAR11/PYR1 (Figure 2B), suggesting that AtMYB44 may specially interact with the 1st subclass of RCARs/PYR1/PYLs [5].

### 2.4. AtMYB44 Negatively Regulates the Expression of ABA-Responsive Gene RAB18

**Figure 3.**
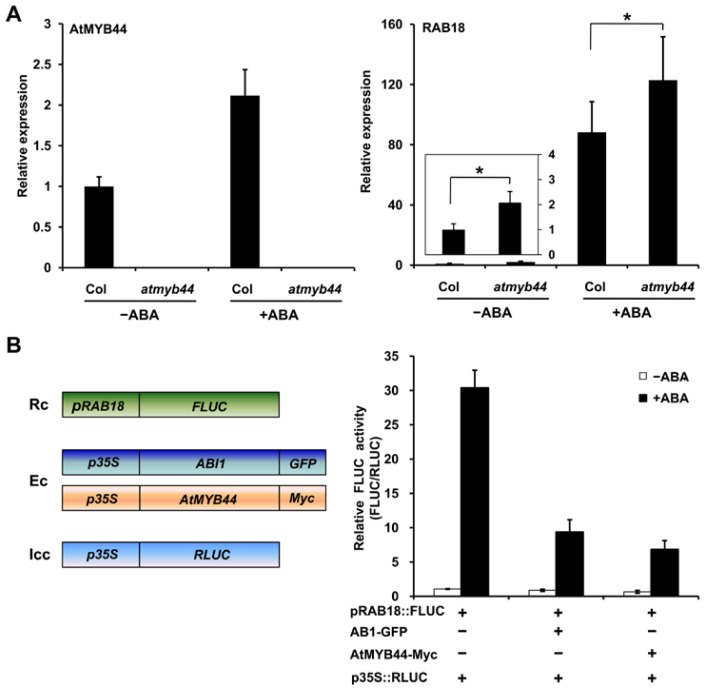
AtMYB44 negatively regulates the expression of ABA-responsive gene *RAB18*. (**A**) Relative expression levels of *AtMYB44* and *RAB18* in wild-type (Col) and *atmyb44* mutant plants were determined by qRT-PCR analysis. 2-week-old seedlings were incubated in 1 × MS liquid medium with ABA (10 μM) or control solvent (DMSO) for 2 h before harvest. *ACTIN2/8* served as an internal control. Error bars indicate SD (*n* = 3). Three independent replicates were analyzed. Asterisks indicate the levels of statistical significance as determined by Student’s *t*-test: * *p* < 0.05 *vs.* Col; and (**B**) The effect of AtMYB44 on ABA-responsive *RAB18* gene expression was analyzed by transactivation assay in *Arabidopsis* protoplasts. **Left panel**: Schematic representation of reporter, effector and internal control constructs used in transactivation assays. Rc indicates Reporter construct; Ec indicates Effector constructs; Icc indicates Internal control construct; **Right panel**: AtMYB44 negatively regulates *RAB18* expression in protoplasts. *pRAB18::FLUC*, *p35S::ABI1*-*GFP*, and *p35S::AtMYB44*-*Myc* plasmids were co-transfected into *Arabidopsis* protoplasts from the wild-type plants as the indicated combinations. *RLUC* was used as an internal control. After transfection, protoplasts were incubated for 5 h under light in the absence (open bars) or presence (filled bars) of 5 μM ABA, and luciferase activity was measured. Values are mean ± SD of three independent experiments.

To understand the significance of the interaction between RCAR1/PYL9 and AtMYB44, we first investigated the role of AtMYB44 in ABA signaling. As mentioned above, there were controversial issues regarding the roles of AtMYB44 in the literature. In order to clarify these issues, we investigated the effect of a knockout mutation of *AtMYB44* on the expression of *RAB18*, an important marker gene in ABA signaling [21,22], using a T-DNA insertion mutant of *AtMYB44* (*atmyb44*) [15–17]. Analysis by quantitative reverse transcription-PCR (qRT-PCR) showed that expression of *RAB18* was slightly enhanced in the *atmyb44* mutant compared to that in wild-type plants, both in the absence and presence of exogenous ABA (Figure 3A), indicating that AtMYB44 negatively regulates the expression of *RAB18*.

To further test the effect of up-regulation of *AtMYB44* on the expression of ABA-responsive gene *RAB18*, we performed transient activation assays with *Arabidopsis* protoplasts using the *RAB18* promoter fused to the *FLUC* gene (*pRAB18::FLUC*) as a reporter. In these assays, the effector genes, *AtMYB44-Myc* or *ABI1-GFP*, were expressed under the control of the 35S promoter and co-transfected together with the reporter construct into the protoplasts. Analysis of LUC activity demonstrated that co-transfection of *pRAB18::FLUC* with *AtMYB44-Myc* resulted in 1.5- and 4-fold reduced *pRAB18::FLUC* expression in the absence and presence of 5 μM ABA, respectively (Figure 3B). This suggests that AtMYB44 acts as a negative regulator in the expression of *RAB18* like ABI1, which is consistent with the result of microarray data published by Jaradat *et al*. [16].

### 2.5. AtMYB44 and ABI1 Compete for Binding to RCAR1/PYL9

**Figure 4.**
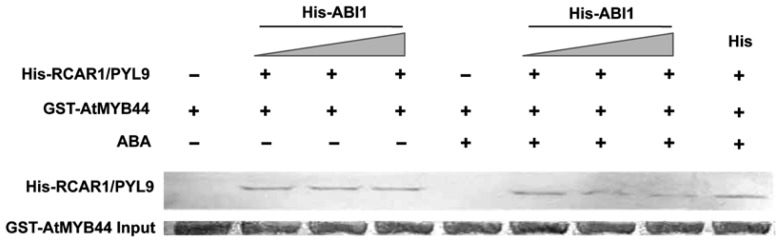
AtMYB44 competes with ABI1 for binding to RCAR1/PYL9 *in vitro*. His-RCAR1/PYL9 was pulled down with GST-AtMYB44 or GST in the absence or presence of His-ABI1 (or His). The gradient indicates increasing amount of His-ABI1. The immune-precipitated fractions were detected by western blotting with anti-His antibody (**upper panel**). GST-AtMYB44 input is shown in the **lower panel**. When indicated, the medium was supplemented with 10 μM ABA. Three independent experiments were performed and similar results were obtained.

To understand how AtMYB44 negatively regulates the expression of ABA-responsive genes, we further focused on the interaction between AtMYB44 and RCAR1/PYL9. Previous reports showed that competition might occur when two proteins interacted with the same protein. For example, the interaction between DELLAs and JAZs affects the binding of MYC2 to JAZs [23], and the interaction between SOS3 and SOS2 affects the binding of ABI2 to SOS2 [24]. Since RCAR1/PYL9 protein functions as an ABA receptor through interacting with PP2Cs and inhibiting their phosphatase activities in the presence of ABA [5,6], the interaction between RCAR1/PYL9 and PP2Cs may affect the binding between RCAR1/PYL9 and AtMYB44. To test this possibility, we performed competitive GST pull-down assays. Recombinant GST or GST-AtMYB44 was used to pull down His-RCAR1/PYL9 in the absence or presence of His-ABI1 (one member of PP2Cs). In the presence of ABA, the interaction between His-RCAR1/PYL9 and GST-AtMYB44 was significantly impaired by an increased amount of His-ABI1 (Figure 4). In another assay, His protein was used as a control instead of His-ABI1. As is shown in Figure 4, His protein did not affect the binding of His-RCAR1/PYL9 and GST-AtMYB44. These results reveal that AtMYB44 and ABI1 compete *in vitro* for binding to RCAR1/PYL9 in the presence of ABA. To test whether other proteins that interact with RCAR1/PYL9 also compete with ABI1 for the binding to RCAR1/PYL9 in the presence of ABA, ArathEULS3 (one protein listed in Table S1) was representatively chosen for research. However, no significant competition between ABI1 and ArathEULS3 was found in our competitive pull down assay (data not shown), suggesting that not all proteins that interact with RCAR1/PYL9 can compete with ABI1 for the binding to RCAR1/PYL9.

### 2.6. AtMYB44 Probably Reduces the Inhibitory Effect of RCAR1/PYL9 on ABI1 Activity in Vitro and in Vivo

**Figure 5.**
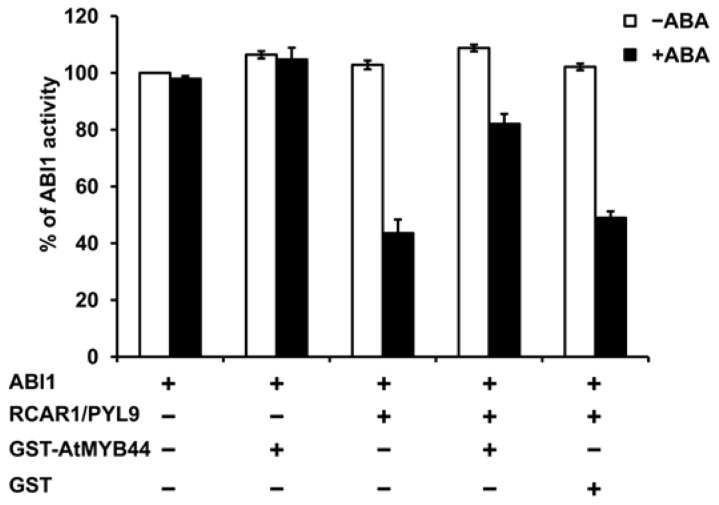
AtMYB44 reduces the inhibitory effect of RCAR1/PYL9 on ABI1 phosphatase activity *in vitro.* ABI1 phosphatase activity was determined using p-nitrophenyl phosphate (pNPP) as the substrate in the absence (open bars) or presence (filled bars) of 10 μM ABA. The concentrations of each protein component were 0.5 μM for ABI1, 5 μM for RCAR1/PYL9, and 10 μM for GST-AtMYB44 or GST. The relative phosphatase activity of each reaction was normalized against the reaction containing substrate and ABI1 (100%). Data are means ± SD from three independent experiments.

Competition between AtMYB44 and ABI1 for binding to RCAR1/PYL9 led us to believe that AtMYB44 could facilitate releasing ABI1 from the ABI1-RCAR1/PYL9 complex through competing with the binding of RCAR1/PYL9 in the presence of ABA. To test this possibility, we examined the effect of AtMYB44 on RCAR1/PYL9-mediated inhibition of ABI1 phosphatase activity using recombinant His-ABI1, GST, GST-AtMYB44, and His-RCAR1/PYL9. As is shown in Figure 5, in the presence of ABA, AtMYB44 proteins alone did not affect the activity of ABI1, but RCAR1/PYL9 proteins alone significantly inhibited its activity as described [5]. When AtMYB44 proteins were added to the reaction mixtures of RCAR1/PYL9 and ABI1, the inhibitory effect of RCAR1/PYL9 on ABI1 phosphatase activity was significantly reduced in the presence of ABA rather than in the absence of ABA (Figure 5), suggesting that AtMYB44 interferes with the ability of RCAR1/PYL9 to suppress ABI1 phosphatase activity through competing for binding to RCAR1/PYL9 with ABI1 in the presence of ABA. To test whether other proteins that interact with RCAR1/PYL9 also affect RCAR1/PYL9-mediated inhibition of ABI1 activity in the presence of ABA, ArathEULS3 (one protein listed in Table S1) was representatively chosen for research. However, no significant effect was found in our PP2C enzyme assay (data not shown), suggesting that not all proteins that interact with RCAR1/PYL9 can reduce the RCAR1/PYL9-mediated inhibition of ABI1 activity.

**Figure 6.**
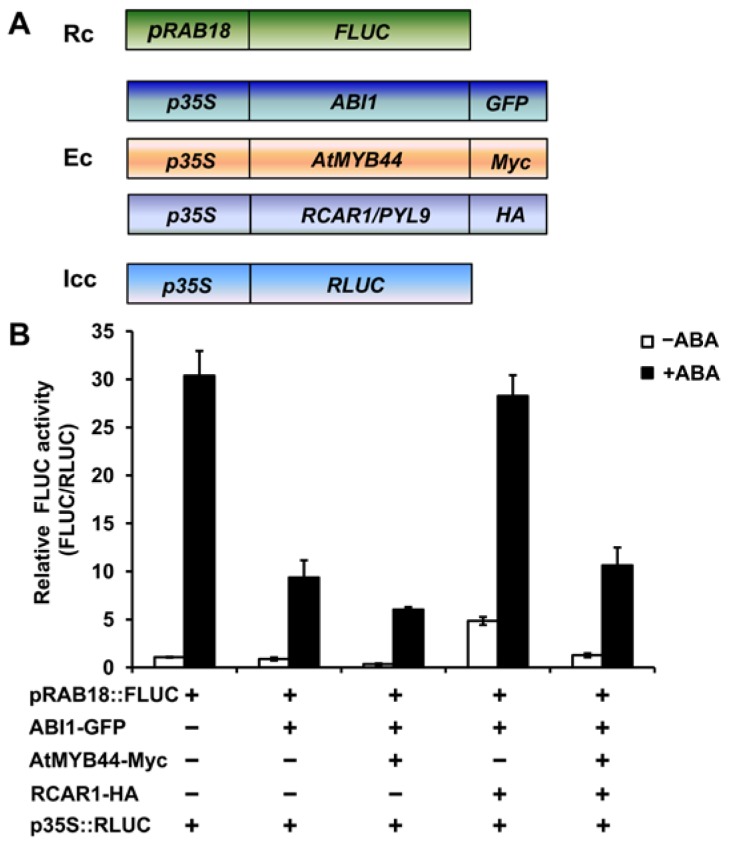
AtMYB44 likely reduces the inhibitory effect of RCAR1/PYL9 on ABI1 activity *in vivo.* (**A**) Schematic representation of reporter, effector and internal control constructs used in transactivation assay. Rc indicates Reporter construct; Ec indicates Effector constructs; Icc indicates Internal control construct; (**B**) AtMYB44 seems to reduce RCAR1/PYL9-mediated inhibition of ABI1 activity in protoplasts. *pRAB18::FLUC*, *p35S::ABI1*-*GFP*, *p35S::RCAR1/PYL9*-*HA*, and *p35S::AtMYB44*-*Myc* plasmids were co-transfected into *Arabidopsis* protoplasts from the wild-type plants as the indicated combinations. *RLUC* was used as an internal control. After transfection, protoplasts were incubated for 5 h under light in the absence (open bars) or presence (filled bars) of 5 μM ABA, and luciferase activity was measured. Values are mean ± SD of three independent replicates.

To determine the role of AtMYB44 in regulating the inhibitory effect of RCAR1/PYL9 on ABI1 phosphatase activity *in vivo*, we performed transient activation assay. Wild-type *Arabidopsis* protoplasts were co-transfected with *pRAB18::FLUC* reporter construct and the indicated combinations of effector constructs encoding *ABI1-GFP*, *RCAR1/PYL9-HA* and *AtMYB44-Myc*. As is shown in Figure 6, transfection of *ABI1* significantly suppressed *pRAB18::LUC* expression in the presence of exogenous 5 μM ABA, consistent with the role of ABI1 as a negative regulator of ABA-responsive genes [25,26]. Addition of *RCAR1/PYL9* weakened the suppression of ABI1 on *pRAB18::LUC* expression, consistent with the result that RCAR1/PYL9 inhibits the phosphatase activity of ABI1 [5]. However, this antagonism is significantly reduced by further co-transfection of *AtMYB44*, indicating that AtMYB44 may also reduce the inhibitory effect of RCAR1/PYL9 on ABI1 *in vivo*. We noted that AtMYB44 alone also slightly increased ABI1-mediated inhibition of *pRAB18::LUC* expression (about 1.55-fold lower than without *AtMYB44* in the presence of ABA), but its effect on antagonizing RCAR1/PYL9-mediated inhibition of ABI1 activity was more significant when *RCAR1/PYL9* was co-expressed, where *pRAB18::LUC* expression was about 2.66-fold lower than without *AtMYB44* in the presence of ABA (Figure 6). This result suggests that AtMYB44 alone increasing ABI1-mediated inhibition of *pRAB18::LUC* expression probably results from its inhibitory effect on endogenous RCAR1/PYL9-mediated inhibition of endogenous ABI1 activity *in vivo*.

## 3. Discussion

**Figure 7.**
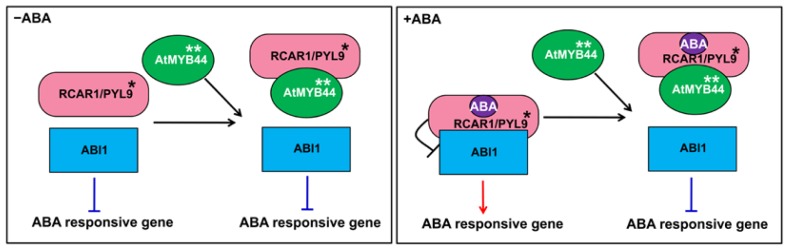
A hypothetical model for the action mechanism of AtMYB44 in ABA signaling. In the absence of ABA, AtMYB44 interacts with RCAR1/PYL9, but does not affect ABI1 activity. Upon ABA perception, AtMYB44 competes with ABI1 for binding to RCAR1/PYL9, releasing the inhibitory of RCAR1/PYL9 on ABI1 activity and thus down-regulating the expression of ABA-responsive genes. “*” indicates that this component may also include RCAR2/PYL7, RCAR3/PYL8 and RCAR4/PYL10. “**” indicates that this component may also include AtMYB70, AtMYB73 and AtMYB77.

AtMYB44 has been reported to participate in ABA signaling in the past few years [15–17], but the topic is controversial. In addition, although the function of AtMYB44 in ABA signaling has been elucidated [15–17], its interacting partners and detailed actions of its mechanism in ABA signaling remains largely unknown. In this study, AtMYB44 was identified as an RCAR1/PYL9-interacting protein via yeast two-hybrid screening. Effect of the functional RCAR1/PYL9-AtMYB44 interaction in ABA signaling was revealed by biochemical and molecular analysis. A hypothetical model for the action mechanism of AtMYB44 in ABA signaling is proposed in Figure 7. In the absence of ABA, RCAR1/PYL9 does not repress the phosphatase activity of ABI1, causing the active ABI1 to suppress the expression of ABA-responsive genes [3,5,13], while AtMYB44 interacts with RCAR1/PYL9 when it is over-expressed. In the presence of ABA, ABA results in the inactivation of ABI1 and the expression of ABA-responsive genes [3,5,13]. However, when *AtMYB44* is over-expressed, it competes with ABI1 for binding to RCAR1/PYL9, partly blocking the inhibitory effect of RCAR1/PYL9 on ABI1 and thus recovering the phosphatase activity of ABI1 to some extent and inhibiting the expression of ABA-responsive genes. Compared with prior work, we first identified the interaction between RCAR1/PYL9 and AtMYB44, further elucidated the significance of the interaction and proposed a possible model to explain how AtMYB44 is involved in ABA signaling. Our work can reasonably support the physiological observations of Jaradat *et al*. [16] and Ma *et al*. [5]. Of course, further genetic analysis still needs to be conducted in future studies to understand the biological roles of RCAR1/PYL9 and AtMYB44 more deeply.

Previous studies showed that all ABA receptors RCARs/PYR1/PYLs interact with PP2Cs and inhibit their phosphatase activities in the presence of ABA [5–7]. In the present study, we demonstrated that RCARs/PYR1/PYLs also interact with AtMYB44, which belongs to the R2R3-MYB transcription factors. Interestingly, unlike the interaction of RCARs/PYR1/PYLs with PP2Cs, RCAR1/PYL9 and RCAR3/PYL8 rather than RCAR8/PYL5 and RCAR11/PYR1 interact with AtMYB44, suggesting that AtMYB44 may specially interact with the 1st subclass of RCARs/PYR1/PYLs [5]. However, this result is contrary to the just published result of Jaradat *et al*., where they reported that AtMYB44 only interacts with RCAR3/PYL8 rather than the other members of PYR/PYL/RCAR family [16]. In fact, we found that RCAR2/PYL7 also interacts with AtMYB44 in the yeast two-hybrid assay (data not shown). These results indicate that although RCARs/PYR1/PYLs all act as ABA receptors, they still have different roles *in planta*. In addition, AtMYB70, AtMYB73 and AtMYB77, three homologous proteins of AtMYB44 in the 22nd subgroup of MYBs also interact with RCAR1/PYL9, but AtMYB2 [27], a member of the 20th subgroup of MYBs does not, indicating that RCAR1/PYL9 specially interacts with the 22nd subgroup of MYBs. This result also suggests that the four members of the 22nd subgroup of MYBs may share similar functions and therefore gene redundancy may exist. It can explain the potential redundancy of AtMYB44 with AtMYB77 in ABA responses and stress signaling [16]. Previous studies showed that the 22nd subgroup of MYBs contain two conserved motifs 22.1 and 22.2 [20]. Thus, it appears that RCAR1/PYL9 may physically bind to one (or both) of the motifs.

As mentioned before, several groups give the opposite opinions concerning the roles of AtMYB44 in ABA signaling [15–17]. In the present study, using qRT-PCR analysis and transient activation assays, we demonstrated that AtMYB44 negatively regulates the expression of ABA-responsive gene *RAB18*, which is consistent with the previous result of microarrays [16]. Nevertheless, *RAB18* expression is only 1.2–1.5 times in *atmyb44* mutant compared with that in the wild-type plants. Our explanation is that *AtMYB44* has three homologous genes in *Arabidopsis*, including *AtMYB70*, *AtMYB73* and *AtMYB77*, and therefore gene redundancy may exist. In fact, gene redundancy indeed exists, since double *atmyb44* × *atmyb77* mutants have more significant ABA-related phenotype than single *atmyb44* and *atmyb77* mutants [16]. In addition, we found that AtMYB70, AtMYB73 and AtMYB77 also interacted with RCAR1/PYL9, further suggesting that members in this family may share similar functions in ABA signaling. Based on our results and previous studies [16], we postulate that AtMYB44 negatively regulates the expression of ABA-responsive genes. However, the mechanism by which AtMYB44 negatively regulates ABA-responsive gene expression was undefined. In this study, we demonstrated that AtMYB44 reduces inhibitory effect of RCAR1/PYL9 on ABI1 phosphatase activity in the presence of ABA. These results allow us to speculate that AtMYB44 negatively regulates the expression of ABA-responsive gene through decreasing the 1st subclass of RCARs/PYR1/PYLs-mediated inhibition of PP2Cs and thereby releasing phosphatase activity of PP2Cs in the presence of ABA. Accordingly, Huang *et al*. [15] and Jaradat *et al*. [16] has demonstrated that AtMYB44 negatively regulates ABA-mediated inhibition of seed germination and drought tolerance using a gain-of-function approach, which is similar to the role of PP2Cs, such as ABI1 [28]. However, in the present study, the interaction between AtMYB44 and RCAR1/PYL9 does not require exogenous ABA supplementation *in vitro* and *in vivo*. And ABA treatment does not affect their interactions in the yeast two-hybrid assay. In contrast, AtMYB44 reduces the inhibition of ABI1 activity by RCAR1/PYL9 *in vitro* only in the presence of ABA. This can be explained through the ABA-dependence of RCAR1/PYL9-mediated inhibition of PP2C. In fact, RCAR1/PYL9 hardly inhibits the phosphatase activity of ABI2 in the absence of exogenous ABA [5]. In addition, we must note that AtMYB44 appears to reduce the inhibitory effect of RCAR1/PYL9 on ABI1 activity both in the presence of ABA and in the absence of ABA *in vivo*, which is not completely consistent with the *in vitro* result. Our explanation is that despite exogenous ABA not being added, endogenous ABA is still at work *in vivo*. In fact, over-expression of *RCAR1/PYL9* in protoplasts activating ABA signaling results from its response to endogenous ABA levels [5]. Further experiments are required to clarify this issue using ABA-deficient mutants [29]. Furthermore, concerning the role of the 22nd subgroup of MYBs, we did not rule out that R2R3 DNA-binding domains are necessary for their functions; perhaps they are necessary for their functions in other plant hormone signaling. In fact, R2R3 DNA-binding domains are necessary for AtMYB77 in auxin response [30]. However, the present study indicates that AtMYB44 interacts with the ABA receptor RCAR1/PYL9 and reduces its inhibitory effect on ABI1 activity to participate in ABA signaling. Accordingly, Fujii *et al*. have reconstituted an ABA signaling pathway and demonstrated that the RCARs/PYR/PYLs, PP2Cs (ABI1 is one of PP2Cs), SnRK2s and ABFs are the only core components to complete the ABA regulation of gene expression [11]. Thus, the other proteins identified as being involved in ABA responses (for example, AtMYB44) may function to modulate the expression and/or activities of one or more of the core components. Considering the role of AtMYB44 in jasmonic acid (JA), salicylic acid (SA), ethylene (ET) and reactive oxygen species (ROS) signaling [31–33], and the interaction between AtMYB44 and RCAR1/PYL9, we are interested in testing if RCAR1/PYL9 is also involved in these signaling in future studies.

In addition, AtMYB44 is regulated by MPK3-mediated phosphorylation [18,19], though it seems that the phosphorylation state of AtMYB44 is not necessary for its function in ABA signaling, since wild-type AtMYB44 can interact with RCAR1/PYL9 and reduce its inhibitory effect on ABI1 phosphatase activity *in vitro* in our present study. Accordingly, DNA binding properties and localization of AtMYB44 have also been shown to be phosphorylation-independent [18]. This can explain why non-phosphorylatable, phosphomimetic and wild-type MYB44 over-expressing lines exhibited similar phenotypes in ABA-mediated inhibition of seed germination [18].

## 4. Experimental Section

### 4.1. Plant Materials and Chemicals

All *Arabidopsis* plants used in this work were in the Columbia (Col-0) background. The *atmyb44* T-DNA insertion mutant (Salk_039074) has been previously described [15–17].

For *in vitro* cultivation, seeds were grown in soil-vermiculite mixtures at 22 °C under 60% humidity with cycles of 16 h light and 8 h dark. For plate culture, seeds were soaked in distilled water for 3 days at 4 °C, then surface sterilized by ethanol and HgCl_2_. After surface sterilization, seeds were sown on the Murashige-Skoog (MS) medium [34] containing 3% sucrose and 0.8% agar, pH 5.9.

All chemicals used were of analytic grade or highest purity and purchased from Sigma (St. Louis, MO, USA), Amresco (Solon, OH, USA) and Chengdu Kelong Chemical Reagent Factory (Chengdu, China).

### 4.2. Plasmid Construction

For yeast two-hybrid assay, the ORFs of *RCAR1/PYL9* and *RCAR3/PYL8* were amplified and cloned into the *Eco*R I-*Pst* I sites of pGBKT7 from Clontech Laboratories, Inc. (Takara Bio Group, Otsu, Japan); the ORFs of *RCAR8/PYL5* and *RCAR11/PYR1* were amplified and cloned into the *Eco*R I-*Bam*H I sites of pGBKT7; the ORFs of *AtMYB44*, *AtMYB70*, *AtMYB73*, *AtMYB77* and *AtMYB2* were amplified and cloned into the *Eco*R I-*Xho* I sites of pGADT7 from Clontech Laboratories, Inc. For GST pull-down assay and PP2C enzyme assay, the ORFs of *RCAR1/PYL9* and *AtMYB44* were amplified and subcloned into pGEX-2T (Amersham, Piscataway, NJ, USA), yielding pGEX-RCAR1/PYL9 (cloning via *Eco*R I and *Xho* I sites) and pGEX-AtMYB44 (cloning via *Eco*R I and *Xho* I sites); the ORFs of *RCAR1/PYL9*, *AtMYB44* and *ABI1* were amplified and subcloned into pET28a (Novagen, Darmstadt, Germany), yielding pET28A-RCAR1/PYL9 (cloning via *Eco*R I and *Xho* I sites), pET28A-AtMYB44 (cloning via *Eco*R I and *Xho* I sites) and pET28A-ABI1 (cloning via *Bam*H I and *Hin*d III sites). For BiFC assay, *RCAR1/PYL9* and *AtMYB44* cDNAs with no stop codon were linked to the truncated YFP genes via *Xba* I and *Sal* I sites in pUC-SPYCE and pUC-SPYNE vectors, respectively. For transient activation assay in protoplasts, *pRAB18::FLUC* reporter plasmid has been described previously [5]. The effector plasmids are all derivatives of pBI221 [35]. The plasmids *p35S::ABI1-GFP* was created as described previously [36]; *p35S::RCAR1/PYL9-HA* and *p35S::AtMYB44-myc* constructs were generated by replacing the glucuronidase gene of pBI221 (via *Xba* I and *Sac* I sites) using the corresponding cDNA fragments; the internal control construct *p35S::RLUC* were produced as described [37]. Primers used in this study are listed in Table S2.

### 4.3. Yeast Two-Hybrid Assay

*Arabidopsis* GAL4 AD/cDNA library was constructed according to Matchmaker cDNA Synthesis protocol from Clontech Laboratories, Inc. Yeast two-hybrid screening was performed as described [38]. The α-galactosidase activity was determined in AH109 yeast report cells as described in the Yeast Protocols Handbook from Clontech Laboratories, Inc. To test the effect of ABA treatment on the interaction between MYBs and RCARs/PYR1/PYLs, 10 μM (+)-ABA (hereafter referred to ABA) was added into the indicated medium.

### 4.4. GST Pull-Down Assay

GST, GST-RCAR1/PYL9, GST-AtMYB44 and His-RCAR1/PYL9, His-AtMYB44, His-ABI1 proteins were expressed in *Escherichia coli* and subsequently purified as described [6,39]. GST pull-down assay was performed as described [38]. For competitive pull-down assays, 5 μg His-RCAR1/PYL9 with 0, 5 or 25 μg His-ABI1, or 25 μg His protein were incubated with immobilized GST-AtMYB44 (5 μg) in protein pull-down incubation buffer 2 (50 mM Tris–HCl, pH 7.5, 100 mM NaCl, 1 mM EDTA, 0.05% β-mercaptoethanol and 0.2% Triton X-100, 10 mM MgCl_2_) at 4 °C for 2 h. Proteins retained on the beads were resolved by SDS-PAGE and detected using anti-GST (Tiangen, Beijing, China) or anti-His antibody (Sigma, St. Louis, MO, USA), respectively. When indicated, 10 μM ABA was added into the buffer.

### 4.5. BiFC Assay

BiFC assay was performed as described [40]. The pUC-SPYNE-AtMYB44 and pUC-SPYCE-RCAR1/PYL9 constructs (containing control plasmids) were introduced into *Arabidopsis* protoplasts, as previously described [41]. Transfected protoplasts were collected 12 h after incubation and stained by DAPI as described [42]. The fluorescence signals of the DAPI-stained protoplasts were then observed and imaged by a Leica confocal laser-scanning microscope (Leica, Buffalo Grove, IL, USA).

### 4.6. RNA Isolation and qRT-PCR Analysis

Total RNAs were extracted from 2-week-old plants of the wild-type and *atmyb44* mutant plants using the RNAiso Plus reagent from Takara (Otsu, Japan). The cDNA was synthesized from 1 μg of total RNAs with PrimeScript RT-PCR reagent Kit of Takara. qRT-PCR was performed using the CFX96 real-time PCR detection system from Bio-Rad Laboratories (Hercules, CA, USA) and SYBR Premix Ex Taq II from Takara (Otsu, Japan). The relative expression levels were calculated using the ΔΔ*C*_t_ method [43], which was normalized against the expression level of *ACTIN2/8*. For all qRT-PCR analyses, three technical replicates and three independent biological repetitions were performed. Statistical significance was assessed by Student’s *t*-test. Values of *p* < 0.05 were considered significant. Primers used for this assay are listed Table S2.

### 4.7. Protoplast Isolation and Transient Activation Assay

Preparation of *Arabidopsis* protoplasts and subsequent transfection of protoplasts were performed as described above. The vector *pRAB18::FLUC* was used at 2.5 μg per transfection, *p35S::AtMYB44*-*Myc*, *p35S::RCAR1/PYL9*-*HA* and *p35S::ABI1*-*GFP* were used at 2 μg per transfection, and *p35S::RLUC* was used at 0.5 μg per transfection. When indicated, 5 μM ABA was added into the incubation buffer. Luciferase activity was measured by an LMax II^384^ luminometer (Molecular Devices, Bad Wildbad, Germany) using the Dual-Luciferase Reporter Assay System from Promega (Madison, WI, USA). Relative *FLUC* activity was calculated by normalizing against the *RLUC* activity, and the data presented were the averages of three biological replicates.

### 4.8. PP2C Enzyme Assay

Proteins used in this assay were expressed and purified as described above. Phosphatase assay using pNPP as substrate was conducted as described [6] with minor modifications. Reaction mixtures (500 μL) contained 50 mM Tris–HCl (pH 8.0), 150 mM NaCl, 10 mM MgCl_2_, 25 mM pNPP, 0.5 μM His-ABI1 with the different combinations of 5 μM His-RCAR1/PYL9 or 10 μM GST-AtMYB44 (or GST) and 0 or 10 μM ABA. The release of PNP was measured at 410 nm, t ~5 s, intervals over 30 min and at 30 °C by a SpectraMAX M2 microplate reader (Molecular Devices, Sunnyvale, CA, USA). The values shown were normalized to the control (ABI1 only; 0 μM ABA) and expressed as % of ABI1 activity.

## 5. Conclusions

In summary, we demonstrate that an R2R3-type MYB transcription factor, AtMYB44, interacts with the ABA receptor RCAR1/PYL9 and likely reduces its inhibitory effect on ABI1 activity *in vitro* and *in vivo*. These results may explain how AtMYB44 participates in ABA signaling.

## Supplementary Information



## Figures and Tables

**Table 1.** RCAR1-interacting R2R3-type MYB transcription factors obtained by yeast two-hybrid assay in *Arabidopsis*.

Clone	Accession Numbers in TAIR a	AA Sequence in Library	AA Sequence in TAIR a	Numbers of Clones
AtMYB44	AT5G67300	48–305	305	3
AtMYB70	AT2G23290	68–144	309	1
AtMYB77	AT3G50060	26–286	301	1

a TAIR, The *Arabidopsis* Information Resource.
