# Supplementary material for: Arabidopsis ABA Receptor RCAR1/PYL9 Interacts with an R2R3-Type MYB Transcription Factor, AtMYB44
Source: Int J Mol Sci. 2014 May 13;15(5):8473–90. doi: 10.3390/ijms15058473 (PMC4057743; doi:10.3390/ijms15058473)
Supplement: Supplementary file 1 [file ijms-15-08473-s001.pdf]

Supplementary Information

Table S1. RCAR1/PYL9-interacting proteins obtained by yeast two-hybrid assay in *Arabidopsis*.

Clone	Accession Numbers in TAIR [a]	AA Sequence in Library	AA Sequence in TAIR [a]	Numbers of Clones
AtMYB44	AT5G67300	48-305	305	3
AtMYB70	AT2G23290	68-144	309	1
AtMYB77	AT3G50060	26-286	301	1
ABI2	AT5G57050	56-227	423	4
Leucine-rich repeat receptor-like protein kinase 902 (RLK902)	AT3G17840	459-647	647	1
UDP-glucosyl transferase 71C5 (UGT71C5)	AT1G07240	323-480	480	1
Gln synthetase (ATGSR2)	AT1G66200	245-414	430	6
Glutamyl-tRNA reductase (HEMA1)	AT1G58290	323-543	543	12
Starch excess1 (SEX1)	AT1G10760	1186-1385	1399	4
ArathEULS3	AT2G39050	160-317	317	2
UBQ10	AT4G05320	32-324	464	2
Indole-3-acetonitrile nitrile hydratase (NIT1)	AT3G44310	192-346	346	1
Voltage dependent anion channel 3 (VDAC3)	AT5G15090	61-274	274	2

[a] TAIR, The *Arabidopsis* Information Resource.

Table S2. Primers used in this article.

Assays	Genes	Primer Sequences (5'–3') [a]
Primers for yeast two-hybrid assay	*RCAR1/PYL9*	F: CTTGAATTCATGGACGGCGTTGAAGGCG<br>R: AACTGCAGTCACTGAGTAATGTCCTGAGAAGCC
	RCAR3/PYL8	F: CCGGAATTCATGGAAGCTAACGGGATTGAGAAC<br>R: AACTGCAGTTAGACTCTCGATTCTGTCGTGTCTTG
	RCAR8/PYL5	F: GGAATTCATGGAAACTTCTCAAAAATATCATACGT<br>R: CGGGATCCTTACAACTTTAGATGAGCCACCCTC
	RCAR11/PYR1	F: CCGGAATTCATGCCTTCGGAGTTAACACC<br>R: CGCGGATCCTCACGTCACCTGAGAACCACTT
	AtMYB44	F: TGGAATTCATGGCTGATAGGATCAAAGGTC<br>R: TCCCTCGAGTTAAACAGTTTCCTAATAATCTCAC
	AtMYB70	F: TGGAATTCATGTCTGGTTCGACCCGG<br>R: AGCCTCGAGCTACTCGATCCTACCTAATCCAAT
	AtMYB73	F: TAGGAATTCATGTCAAACCCGACCCGT<br>R: ATCCTCGAGCTGAAACTTTTATTTTCCCGAG
	AtMYB77	F: TGGAATTCATGGCTGATCGTGTTAAAGGT<br>R: TACCTCGAGCTACTCAACCTTAGGTGTTATTACTC
	AtMYB2	F: TGGAATTCATGGAAGATTACGAGCGAATA<br>R: TACCTCGAGTTAATTATACGAATACGATGTCGTA
Primers for GST pull-down assay and PP2C enzyme assay	*RCAR1/PYL9*	F: CTTGAATTCATGGACGGCGTTGAAGGCG<br>R: TCCCTCGAGTCACTGAGTAATGTCCTGAGAAGCC
	AtMYB44	F: TGGAATTCATGGCTGATAGGATCAAAGGTC<br>R: TCCCTCGAGTTAAACAGTTTCCTAATAATCTCAC
	ABI1	F: TTAGGATCCATGGAGGAAGTATCTCCGGC<br>R: CTCAAGCTTTCAGTTCAAGGGTTTGCTC
Primers for BIFC assay	*RCAR1/PYL9*	F: TGTCTAGAATGGACGGCGTTGAAGGCG<br>R: TCCGTCGACCTGAGTAATGTCCTGAGAAGCC
	AtMYB44	F: TGTCTAGAATGGCTGATAGGATCAAAGGTC<br>R: TCCGTCGACCTCGATTCTCCCAACTCCAATTTG
Primers for qRT-PCR analysis	*AtMYB44*	F: GTTGTGCTACCGCTTCCTATCG<br>R: ATGTAACTCCTCACTTCCGCCT
	RAB18	F: ATGGCGTCTTACCAGAACCGT<br>R: CCAGATCCGGAGCGGTGAAGC
	ACTIN2/8	F: GGTAACATTGTGCTCAGTGGTGG<br>R: AACGACCTTAATCTTCATGCTGC
Primers for transient activation assay	*RCAR1/PYL9*	F: TGTCTAGAATGGACGGCGTTGAAGGCG<br>R: CGAGCTCTCAAGCGTAATCTGGAACATCGTATGGGTACTGAGTAATGTCCTG
	AtMYB44	F: TGTCTAGAATGGCTGATAGGATCAAAGGTC<br>R:CGAGCTCTCAATCCTCCTCAGAAATCAACTTTTGCTCCTCGATTCTCCCAAC

[a] Restriction sites are marked in red, and nucleotide sequences of HA-tag or Myc-tag are marked in blue. F indicates forward primer, and R indicates reverse primer.
